# Patellar development after patella instability and early reduction in growing rabbits

**DOI:** 10.1186/s12891-023-06183-6

**Published:** 2023-01-30

**Authors:** Weifeng Li, Qian Wang, Haiying Wang, Zipeng Zhang, Shunyi Wang

**Affiliations:** 1 Department of Orthopaedic Surgery, Baoding No 1 Central Hospital, No. 320 Changcheng Street, Baoding, 071000 Hebei People’s Republic of China; 2 The First Department of Operating Room, Baoding No 1 Central Hospital, No. 320 Changcheng Street, Baoding, 071000 Hebei People’s Republic of China

**Keywords:** Knee, Patella instability, Reduction, CT, Rabbit

## Abstract

**Background:**

Patella-shaped disorder has been considered as a predisposing factor for patella instability. But the influence of early patella reduction for patellar development remains unclear. This study aimed to evaluate whether early operation in patella instability could improve patella morphology in growing rabbits.

**Methods:**

Fifty rabbits (1-month-old) were included in the study. The control group underwent no surgical procedures. The two experimental groups (reduction group and non-reduced group), underwent medial soft tissue restraint release surgery. The reduction group, rabbits underwent the medial soft tissue sutura surgery in order to stabilize the patella 2 months after release surgery. The non-reduced group, rabbits did not undergo suture surgery. Computed Tomography (CT) scans analysis in two experimental endpoints (2, 5 months after release surgery) were selected to evaluate the transverse diameter, thickness, Wiberg index and Wiberg angle. Gross observation was conducted to assess morphological changes of the patella.

**Results:**

CT scans showed significant difference in the mean transverse diameter, Wiberg angle between the two experimental groups and the control group 2 months after release surgery. 5 months after release surgery, the indices of patella were found no statistically difference in the reduction group versus the control group. However, the transverse diameter, Wiberg angle in the non-reduced group were significantly differences than that in the reduction group (*P* < 0.05). Gross observation showed a flattened articular surface of the patella in the non-reduced group.

**Conclusions:**

The results indicated that patella instability may lead to patella-shaped disorder, showing a flattened morphology. Early patella reduction can improve the patella morphology in growing rabbits.

## Introduction

The biomechanics of the patellofemoral joint is a involved subject, which is contained by the articulation between the patella and the trochlear groove. The patella is located at the distal end of the quadriceps. Wibeeg et al. [1] presented an anatomical morphology of the patella that showed a medial crest crossing over the articular portion of the patella and defined a medial and a lateral articular facet. The patella plays an important role in the function of the knee joint. It increases the biomechanical lever arm and improves the effective extension capacity of the knee. In addition, it has the effect of preventing excessive friction between bone and tendon [2]. The patella is in contact with and limited by the femoral trochlea during flexion and extension of the knee.

For patella instability, previous studies have shown that the incidence of patella instability is higher in children and teenagers, especially in girls. The estimated annual incidence of primary patella dislocation in children aged 9–15 years is 107 per 100,000, compared to 7 per 100,000 in all age groups [3, 4]. Patella instability not only eventually lead to patellofemoral arthritis, but also affect the development of the patellofemoral joint.

Numerous anatomic factors have been associated with patella instability, including trochlear dysplasia, patella alta, rotational deformities, patella shape and increased tibial tubercle-trochlear groove (TT-TG) distance [5–10]. Among the factors, Trochlear dysplasia has been described as a risk factor for patella instability in previous studies [6–8]. A magnetic resonance imaging study showed that patients with patella instability exhibit a flatter trochlear groove than those without instability [11]. The influence of the patella position on the development of the femoral trochlea has been continuously studied. Li [12] and Huri [13] found that early patella dislocation could occur trochlear dysplasia in growing rabbits. Wang et al. [14] showed that early relocation of the patella can prevent the development of trochlear dysplasia in growing rabbits. Benoit et al. [15] reported that children with patella instability (< 10 years) showed significant improvement in the sulcus angle after a patellar stabilisation surgery. These studies indicate that femoral trochlea dysplasia could be caused by instability of the patella.

Recent studies have proven that patella dislocation not only results in trochlear dysplasia but also affect the development of the patella. Panni AS et al. [10] demonstrated that patella shape disorder also play a role in patella instability. Servien et al. [16] showed that in patients with patella dislocation often exist a short patellarapex and a hypoplastic medial border. Niu et al. [17] recently reported that patella dysplasia can be caused by patella instability in rabbits. Although the patella articulates with the femoral trochlea, the influence of early patella reduction for patellar development remains unclear. Although the trochlear morphology can be improved by early surgical correction with patella dislocation, few studies have described the role of patella reduction on the development of patella.

The genesis and growth of the patellofemoral joint is similar to that of the hip joint. Dysplastic acetabulum can be remodelled by mechanical stress stimulation has been shown. Considering the similarities between the patellofemoral joint and the hip joint, patella morphology may be improved by early patella reduction. The aim of the study was to determine whether early patella reduction could improve the patella morphology in growing rabbits. We hypothesize that early patella instability may lead to patella-shaped disorder, and early patella reduction can improve the patella morphology.

## Materials and methods

### Study design

The experimental protocol was approved by the local Animal Care and Use Committee.

Fifty 1-month-old female New Zealand healthy white rabbits, weighing between 320 and 420 g (provided by the local Animal Center) were included in the study. The control group consisted of 50 right knees, which underwent no surgical procedures. The two experimental groups comprised 50 left knees, which were performed patella instability surgery (medial soft tissue restraint release). In order to stabilize the patella, the reduction group (N = 25 knees) underwent medial soft tissue sutura 2 months after release surgery. The non-reduced group (N = 25 knees) underwent no suture surgery. All procedures executed involving animals were under the Western University’s Animal Care and Use Guidelines (London, Ontario, Canada) [18].

### Surgical procedures

**Fig. 1:**
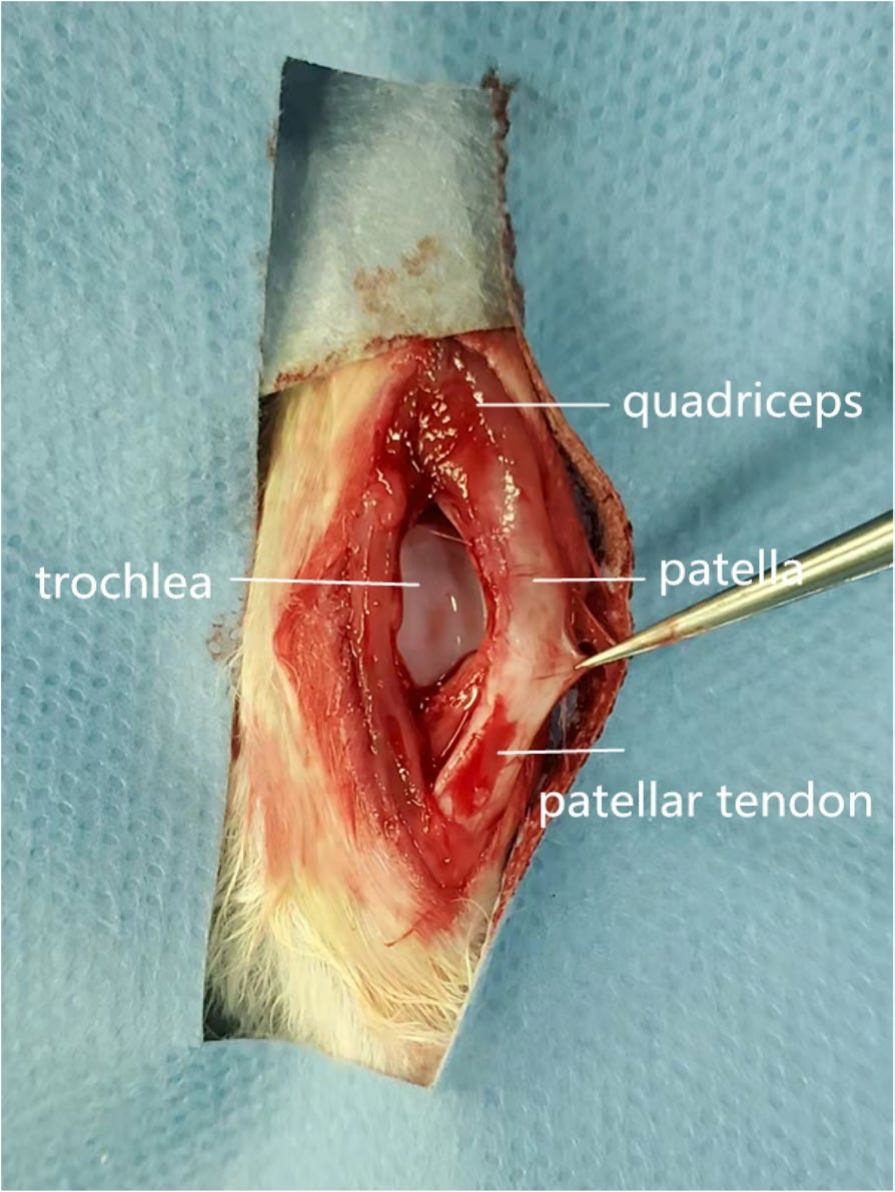
The picture during operative. The medial soft tissue restraint was released. Patella was moved laterally and femoral trochlear could be noticed

The surgical protocol for making patella instability models of growing rabbits have been proved by previous studies [14, 17]. First, the rabbits were intravenous anesthetized with ketamine (20 mg/kg) and xylazine (5 mg/kg) through the ear vein. The knee was shaved and prepped in a sterile following standard procedure pre-operation. Next, a 4-cm longitudinal incision was preferred on the knee joint in the two experimental group, the soft tissue of medial retinaculum and the joint capsule were dissected and exposed. Then, a 2.5-cm longitudinal incision was made along the medial border of the patella to incise the medial retinaculum and joint capsule. The state of patella instability was found intraoperatively (Fig. 1). The patella was in dislocated state when the knee was flexed, and the patella returned to the relatively normal location when the knee was straighted. Finally, the incision was irrigated and sutured without reconstruction of medial retinaculum. 2 months after medial soft tissue restraint release surgery, the reduction group underwent patellar reduction via the original longitudinal incision. The skin and soft tissue were dissected and the medial patellar retinaculum were exposed. Then, the medial patellar retinaculum was sutured, keeping patella in the normal trochlear groove. Finally, the incision was irrigated and sutured in layers. Prudence was performed to avoid damage the articular cartilage. All rabbits were raised under the same conditions (food, water and individual steel cage (310 × 550 × 320 mm)) and allowed free activity in cages. Skeletal maturation of rabbits is complete at 6 months of age [19], so all the rabbits were euthanized (by intravenous injection of pentobarbital, 100 mg/kg) at 5 months after soft tissue release surgery.

### CT measurements

**Fig. 2:**
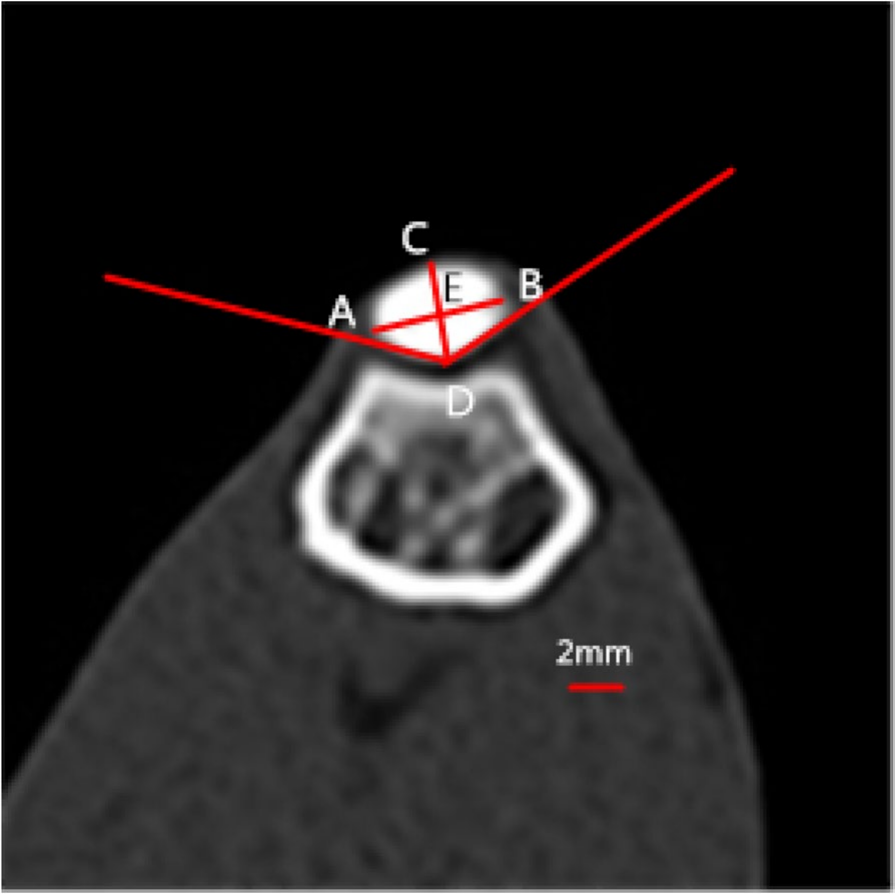
AB is defined as transverse diameter; CD is defined as thickness; length of BE/length of AB is defined as Wiberg index; angle ∠D is defined as Wiberg angle

CT scans of the rabbits were performed immediately after the release operation, 2 and 5 months after release surgery using a 16-slice CT scanner (SOMATOM Sensation 16; Siemens Medical Solutions, Erlangen, Germany). All CT images were captured on the axial plane, which is the optimal position to observe and measure the patellofemoral joint. The measurements were performed by RadiAnt-DICOM software (Medixant Ltd, Poznan, Poland) which has a 0.1°and 0.01 mm measuring accuracy (Fig. 2). The CT slice image with the widest diameter of the patella was acquired for the indice measurements in the transverse plane. The transverse diameter (AB) of the patella was described by Stäubli [20], which was defined as the length between the most medial edge (A) and the most lateral edge (B) of the patella. The posterior patellar edge farthest from the baseline (AB) was defined as point D. The thickness of the patella was measured between the most anterior point (C) of the patella and the most posterior point (D) of the patellar edge, and line CD vertical to the baseline (AB). The insertion between line AB and line CD was defined as point E. The Wiberg index was defined as the ratio of the length of BE to the length of AB. For the Wiberg angle (∠D) measurement, as described by Fucentese [21], which was defined as between the slopes of the medial patella and the lateral patella. The trochlear groove measurements were taken from the CT slice image through the intersection of lower femoral physis line with posterior cortex [14]. CT measurements included both the angle and depth of the femoral trochlea. The femoral trochlear angle was defined as the angle between the deepest point of trochlear sulcus and peak point of the bilateral condyle. Measuring methods of the trochlear depth was based on the published studies on the tomographic images which were defined as the vertical distance from the deepest point of trochlear sulcus to the line connecting the peak point of the bilateral condyle. All measurements were taken blindly by two independent researchers. To determine the intra-observer variation, one researcher repeated the observations at 7 days after the first measurement.

### Cross observation

The rabbit patella dislocation test was performed at 2 and 5 months after release surgery. It was performed by pushing the patella outward with the thumb, at 30 degrees of knee flexion. A positive patella dislocation test was indicated if the patella was dislocated. CT scans were performed 5 months after release surgery. Subsequently, the rabbits were euthanized, The skin and soft tissue were carefully removed and the patella and trochlea were dissected. Morphological differences were taken to observed and recorded.

### Statistical analysis

SPSS statistical software (version 22.0; SPSS, IL, USA) was performed for data analyse. The data were compared with Student’s t test between the control and experimental groups immediately after release operation. Dunnett’s multiple-comparisons was used to evaluate the transvers diameter, thickness, Wiberg index, and Wiberg angle of patella among the three groups at each time point. *P* < 0.05 were defined as the threshold for statistical significance. The results values were expressed by Mean ± SD. The inter-and intraobserver reliabilities were then determined by calculating intra-class correlation coefficients (ICCs).

## Results

### CT measurement

**Fig. 3:**
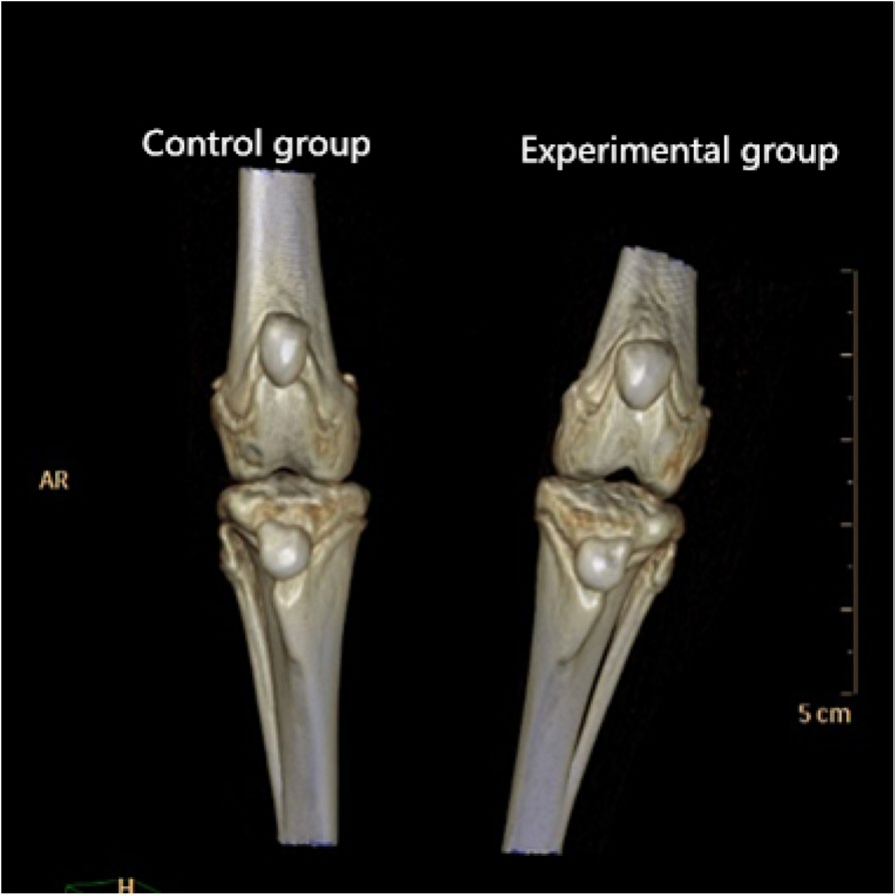
Three-dimensional reconstructed CT diagram of the patella in the surgically treated experimental group and the non-surgically treated control group, 2 months after release surgery. The transverse diameter of the patella is longer in the experimental group (right) than in the control group (left)

In the study, the transverse diameter, thickness, Wiberg index and Wiberg angle were measured immediately after release surgery, and the values were not significantly different among the three groups (Table 1). However, 2 months after release surgery, the mean transverse diameter in the control group was 5.50 ± 0.38 mm, the mean transverse diameter in the non-reduced group was 7.03 ± 0.36 mm and the mean transverse diameter in the reduction group was 7.01 ± 0.33 mm. The mean Wiberg angle showed the greater difference: 128.6 ± 5.3°in the control group versus 136.4 ± 6.7°in the non-reduced group and 136.5 ± 6.5°in the reduction group. The experimental groups had longer transverse diameter and larger Wiberg angle than the control group (Fig. 3). Furthermore, 5 months after release surgery, the mean transverse diameter in non-reduced group was 7.86 ± 0.64 mm, while that in reduction group was 6.35 ± 0.46 mm; the difference between the two groups was statistically signifcant (*P *< 0.05) (Table 2). 2 and 5 months after release surgery, the mean thickness of the patella was not significantly different among the three groups (Table 3). Changes in Wiberg index (Table 4) showed the same pattern as thickness. However, 5 months after release surgery, the mean Wiberg angle in non-reduced group was 138.5 ± 6.4°, while that in reduction group was 129.8 ± 4.5°; the difference between the two groups was statistically signifcant (*P* < 0.05) (Table 5). For patella dislocation test, the rate of positive patella dislocation test was 100% (50/50) in the two experimental groups 2 months after release surgery. The rate of positive patella dislocation test was 96% (24/25) in the non-reduced group while that in the reduced group was 0 (0/25) 5 months after release surgery. For femoral trochlea, the mean femoral trochlear angle was 135.9° ± 5.2°in non-reduced group 5 months after release surgery, the reduction group was 125.2° ± 3.6°. The femoral trochlear angle of the two groups were significantly different (*P* < 0.05). The mean trochlear depth was 1.31 ± 0.03 mm in non-reduced group 5 months after release surgery, while that in reduction group was 1.52 ± 0.05 mm. The trochlear depth of the two groups were significantly different (*P* < 0.05). On the other hand, five indices about the patella and two indices about the trochlea between the reduction group and the control group were not statistically different 5 months after release surgery. As a result, the reduction group had normal patella and trochlea morphology. The inter-and intraobserver correlation coefficients were showed in Table 6.

**Table 1:** Measurements immediately after release operation (X ± SD)

Indexes group	Control group	Non-reduced group	Reduction
Mean AB(mm)	3.81 ± 0.24	3.80 ± 0.27	3.79 ± 0.25
Mean CD(mm)	2.82 ± 0.37	2.85 ± 0.36	2.86 ± 0.32
Wiberg index	0.53 ± 0.03	0. 52 ± 0.03	0. 53 ± 0.02
Mean Wiberg angle(°)	130.8 ± 6.6	131.5 ± 6.2	130.3 ± 5.6

*AB* transverse diameter of the patella, *CD* thickness of the patella

**Table 2:** The measurements of transverse diameter (mm) in the three groups (X ± SD)

	Non-reduced group	Reduction group	Control
Two months	7.03 ± 0.36*	7.01 ± 0.33*	5.50 ± 0.38
Five months	7.86 ± 0.64*&	6.35 ± 0.46	6.27 ± 0.39

^*^ Significant difference compared with the control group (*p* < 0.05)

&Significant difference compared with the reduction group group (*p* < 0.05)

**Table 3:** The measurements of thickness (mm) in the three groups (X ± SD)

	Non-reduced group	Reduction group	Control
Two months	3.81 ± 0.28	3.82 ± 0.24	3.85 ± 0.32
Five months	4.23 ± 0.44	4.18 ± 0.36	4.11 ± 0.32

**Table 4:** The measurements of Wiberg index in the three groups (X ± SD)

	Non-reduced group	Reduction group	Control
Two months	0.49 ± 0.05	0.49 ± 0.04	0.48 0.06 ±
Five months	0.51 ± 0.04	0.50 ± 0.05	0.50 0.03 ±

**Table 5:** The measurements of Wiberg angle (°) in the three groups (X ± SD)

	Non-reduced group	Reduction group	Control
Two months	136.4 ± 6.7*	136.5 ± 6.5*	128.6 5.3 ±
Five months	138.5 ± 6.4*&	129.8 ± 4.5	129.2 5.0 ±

^*^ Significant difference compared with the control group (*p* < 0.05)

&Significant difference compared with the reduction group group (*p* < 0.05)

**Table 6:** Intra-observer and inter-observer agreement of geometric measurements with 95% confidence intervals(CI)

Measurement	Intraobserver		Interobserver	
	ICC	95% CI	ICC	95% CI
RG-AB	0.776	0.726 to 0.857	0.741	0.699 to 0.822
RG-CD	0.813	0.725 to 0.865	0.768	0.635 to 0.858
RG-WI	0.873	0.780 to 0.904	0.863	0.801 to 0.932
RG-WA	0.736	0.690 to 0.852	0.728	0.653 to 0.835
NRG-AB	0.764	0.731 to 0.880	0.745	0.702 to 0.873
NRG-CD	0.863	0.798 to 0.937	0.856	0.808 to 0.936
NRG-WI	0.830	0.784 to 0.920	0.828	0.757 to 0.916
NRG-WA	0.736	0.681 to 0.805	0.732	0.631 to 0.803
CG-AB	0.872	0.756 to 0.906	0.855	0.808 to 0.920
CG-CD	0.833	0.774 to 0.912	0.820	0.714 to 0.916
CG-WI	0.766	0.701 to 0.845	0.739	0.705 to 0.858
CG-WA	0.828	0.752 to 0.889	0.810	0.730 to 0.869

*ICC* intra-class correlation coefficient, *RG* reduction group, *AB* transverse diameter of the patella, *CD* thickness of the patella, *WI* Wiberg index, *WA* Wiberg angle, *NRG* non-reduced group, *CG* control group

### Gross observation

**Fig. 4:**
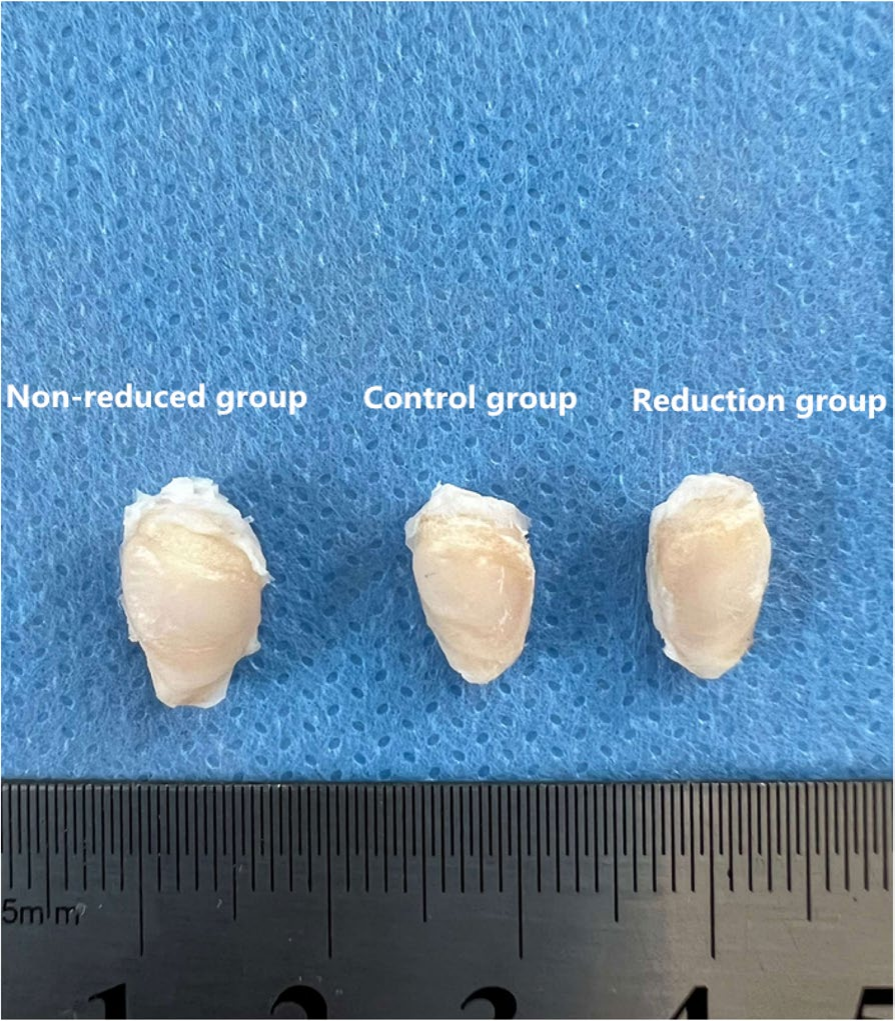
Gross anatomy of the patella: 5 months after release surgery of the non-reduced group (left), 5 months after release surgery of the control group (middle), 5 months after release surgery of the reduction group (right). The articular surfaces of all three groups were smooth. However, the articular surface of the patella was flatter in the non-reduced group (left) than in the control group (middle) and the reduction group (right). The transverse diameter of the patella was longer in the non-reduced group (left) than in the control group (middle) and the reduction group (right)

5 months after release surgery, the non-reduced group had wider and more flattened patella than the other groups, and the femoral trochlea in the non-reduced group was shallower compared to in the other groups. In the reduction group, the patella and trochlea morphology appeared no morphological changes compared with the control group (Fig. 4).

## Discussion

The key findings in the study were that patella instability may lead to patella-shaped disorder in growing rabbits, and early patella reduction could improve patella morphology.

Studies the mechanism elucidation and pathological changes during trochlear dysplasia have continued to be published. Huri [13] and Kaymaz [22] had demonstrated that after early patella dislocation or patella alta, a higher trochlear groove angle and lower trochlear depth were found in growing rabbits. The higher trochlear groove angle and lower trochlear depth in the experimental groups in these studies described above corresponded to a shallower trochlea in the non-reduced group in the present study. Wang et al. [14] had confirmed that after patella subluxation can lead to trochlear dysplasia and that early relocation of the patella can prevent trochlear dysplasia. Fu et al. [23] recently found that the morphology of the femoral trochlea may improve after surgical correction of patella instability in children. The present study produced similar results to the above studies. In the reduction group, no significant change in trochlea morphology compared to the control group.

Patella morphological characteristics were a major aspect of the study and yielded interesting results. In recent studies, it had been demonstrated that patients with trochlear dysplasia also present with patella dysplasia [15, 24]. Fucentese et al. [22] found that patients with trochlear dysplasia had a smaller medial facet and a higher prevalence of type II and type III patella compared to controls. Panni et al. [10] found an association between patellar morphology type and femoral trochlear dysplasia grade III and a correlation between patella tilt and patella shape. Li et al. [25] reported that patients with trochlear dysplasia had a patella of smaller width, thinner thickness, more flattened articular facet. Yılmaz et al. [26] examined 20 children with patellofemoral dislocation compared to an age-matched healthy control group and found that the mean length and width of the patella in the two groups were significantly different, the patella in the two groups were significantly different. Niu et al. [17] studied 40 knees from 20 rabbits that were divided into an experimental group (underwent a medial soft tissue restraint release) and control group (no surgical interventions). The study demonstrated that the shape and articular surface of the patella became more flattened after patella dislocation in experimental group.

From the abovementioned studies, patella morphology was believed to be correlated with patella stability. However, the potential influence of patella reduction have not been described in growing rabbits. In the study, it was established that patella instability can cause the change in patella morphology and that early patella reduction can improve the patella morphology. The importance of patella stability for patella development was emphasised.

On the basis of the previously animal studies, we modified the model of patella dislocation, and used a model of patella instability similar to that of humans. The patella was in dislocated state and the trochlear groove was seen when the knee was flexed, and the patella returned to the relatively normal location when the knee was straighted. We believe that the animal model can better reflect the patella morphology changes in human knee that are related to patella instability. In the present study, the transverse diameter and the Wiberg angle were statistically signifcant, while the thickness and Wiberg index were not significantly different between the non-reduced group and the reduction group, which indicates that patella instability may affect patellar diameter but not patellar thickness. Patella morphology was altered significantly after patella instability, which indicates that patella shape is effected by epigenetic factors. This is consistent with the results of Niu [17]. In the study, there was no significant differences in the five indices of patella between the reduction and control group, which is similar to the results of Wang [14] on the femoral trochlea. Therefore, the conclusion that early patella reduction can improve patella morphology was confirmed by authors. The patellofemoral joint development, including the patella and femoral trochlear, could be influenced by patella stability.

Of these measures, the transverse diameter of patella and Wiberg angle are probably the most important to indicate that the patella has been stabilized. These findings may develop pathology and etiology of patella instability, and emphasize the importance of the early effective treatments for patella instability.

Although the cause of patellar dysplasia is not clear, the two possible reasons should be attention: absence of patellar mechanical stress and the tension of medial patellofemoral ligament. Grelsamer RP et al. [27] indicated that mechanical stress is a important factor affecting bone growing development, which can stimulates the growth and remodelling of the patella and femoral trochlea during functional movement [28]. Considering the similarities between the patellofemoral joint and the hip joint. Abnormal soft-tissue laxity has been established in the developmental dysplasia of the hip (DDH) population and its role described in the aetiology of hip instability. By the same token, soft-tissue laxity can influence the stability of the patella in the trochlea and potentiate abnormal development of the patella and trochlea if the patella does not track normally. It is plausible that early diagnosis in the growing period would allow for intervention in a fashion similar to DDH. Thus, the patella morphology could be remodeled, when early medial soft tissue restraint tension returns to normal and the patella reduction. The findings of the animal studies indicate that morphological changes of patella during childhood should be early intervention to avoid patella dysplasia in patellofemoral joints in the following years.

This study has several limitations. First, rabbit models rather than human bodily specimens were used in the study. The anatomy and growing period of animals do not always match the clinical situation in human. Second, the number of immature rabbits was narrow, even though it showed statistical differences. More animals would have been required to more accurately assess for the study. Also, changes in cartilage, subchondral bone and molecular level should be researched in the future.

## Conclusions

In conclusion, the study demonstrated that patella instability in growing rabbits can result in patella-shaped disorder, and early patella reduction can improve the patella morphology. Clinically, morphological changes of the patella in childhood with patella instability should be taken seriously and early intervention may be important.

## Data Availability

The detailed data and materials of this study were available from the corresponding author through emails on reasonable request.

## Abbreviations

TT-TG: Tibial tubercle-trochlear groove
CT: Computed Tomography
ICCs: Intra-class correlation coefficients
RG: Reduction group
AB: Transverse diameter of the patella
CD: Thickness of the patella
WI: Wiberg index
WA: Wiberg angle
DDH: Developmental dysplasia of the hip
NRG: Non-reduced group
CG: Control group
